# 

**Published:** 1889-09

**Authors:** 


$2.00 A YEAR IN ADVANCE.

WHOLE NUMBER, CCCXXXVIII.

New Series.

September, 1889.

VOL. XXIX. No. 2.

INDEX S.G.

### BUFFALO MEDICAL AND SURGICAL JOURNAL.

Established 1845 by AUSTIN FLINT, M. D.

PUBLISHED MONTHLY.

**Editors:**

THOS. LOTHROP, M. D.  W. W. POTTER, M. D.

**Associate Editors:**

FRANK HAMILTON POTTER, M. D.  JAMES WRIGHT PUTNAM, M. D.

WILLIAM C. KRAUSS, M. D.  JOHN PARMENTER, M. D.

SCRIPTA FERUNT ANNOS

BUFFALO:

1889.

Entered at the Post-Office at Buffalo, N. Y., as Second-class Mail Matter.

*Times Printing House, Buffalo, N. Y.*

